# Obeticholic Acid Improves Cholestasis, Liver Fibrosis, and Liver Function in Patients with Primary Biliary Cholangitis with Inadequate Response to Ursodeoxycholic Acid

**DOI:** 10.3390/jpm15030079

**Published:** 2025-02-21

**Authors:** Matthias Buechter, Paul Manka, Kerem Bulut, Guido Gerken, Alisan Kahraman

**Affiliations:** 1 Department of Gastroenterology and Hepatology, University Clinic of Essen, University of Duisburg-Essen, 45147 Essen, Germany; m.buechter@kkimk.de (M.B.); guido@clausgerken.de (G.G.); 2 Department of Gastroenterology and Hepatology, St. Elisabeth Hospital, 58638 Iserlohn, Germany; 3 Department of Internal Medicine, University Hospital Knappschaftskrankenhaus Bochum, Ruhr University Bochum, 44892 Bochum, Germany; paul.manka@ruhr-uni-bochum.de; 4 Clinic for Internal Medicine and Gastroenterology, St. Clemens-Hospital Geldern, 47608 Geldern, Germany; k.bulut@clemens-hospital.de; 5 Department of Gastroenterology and Hepatology, Helios Clinic, 42549 Velbert, Germany; 6 Department of Gastroenterology and Hepatology, Max Grundig Clinic, 77815 Bühl, Germany

**Keywords:** FibroScan, LiMAx, obeticholic acid, PBC, primary biliary cholangitis

## Abstract

**Background and Aims:** Primary biliary cholangitis (PBC) leads to the slow, progressive destruction of the small bile ducts with consecutive cholestasis and intrahepatic cholangitis. If this disease remains untreated, liver parenchyma will be damaged resulting in fibrosis and end-stage liver disease with the need for transplantation. The approval of the Farnesoid X receptor agonist obeticholic acid (Ocaliva; OCA) in early 2017 expanded the drug therapy options of PBC, which previously consisted primarily of the administration of ursodeoxycholic acid (UDCA). **Patients and Methods:** Included in our prospective pilot study were 16 patients with a confirmed diagnosis of PBC who were treated with an add-on therapy with OCA (5 mg/d). None of the patients had an overlap to autoimmune hepatitis. Patients were investigated between 09/2022 and 09/2023. **Results:** The majority of patients was female (15/16, 93.75%), and the mean age was 57.63 ± 9.59 (43–77) years. OCA treatment led to a statistically significant decrease in aspartate aminotransferase (AST; AST baseline: 38.50 [26.25; 50.00] IU/L vs. AST 6-month follow-up: 23.50 [21.50; 44.25] IU/L, *p* = 0.0012), alanine aminotransferase (ALT; ALT baseline: 55.50 [28.75; 97.00] IU/L vs. ALT 6-month follow-up: 36.50 [28.00; 57.25] IU/L, *p* = 0.0035), and gamma-glutamyl transferase (GGT; GGT baseline: 168.00 [100.30; 328.50] IU/L vs. GGT 6-month follow-up: 88.00 [44.50; 259.80] IU/L, *p* = 0.0063), while the decrease in alkaline phosphatase (AP) was not statistically significant (AP baseline: 197.00 [170.00; 253.30] IU/L vs. AP 6-month follow-up: 196.00 [134.00; 227.00] IU/L, *p* = 0.0915). In addition, liver stiffness measurement (LSM) showed a statistically significant decrease after six months of treatment with OCA (LSM baseline: 7.85 [5.55; 10.13] kPa vs. LSM 6-month follow-up: 5.95 [4.55; 8.225] kPa, *p* = 0.0001). However, the increase in enzymatic liver function measured by LiMAx failed to reach statistical significance, but showed a positive trend (LiMAx baseline: 402.50 [341.50; 469.80] μg/kg/h vs. LiMAx 6-month follow-up: 452.50 [412.50; 562.00] μg/kg/h, *p* = 0.0625). In none of our patients did therapy with obeticholic acid have to be stopped due to pruritus or poor tolerability. **Conclusions:** In patients with PBC without adequate response to UDCA, OCA is a promising alternative, which in our group of 16 patients led to a significant improvement of liver enzymes, the amelioration of liver fibrosis, and an increase in liver function capacity in a short-term clinical course.

## 1. Introduction

Primary biliary cholangitis (PBC) is a chronic, autoimmune, and inflammatory liver disease characterized by destruction of the small intrahepatic bile ducts [1,2]. Since PBC is considered as an autoimmune disease, there is high gender specificity (approximately 90% of affected individuals are female), positivity to disease-specific anti-mitochondrial antibodies (AMAs) accompanied by elevated immunoglobulin M (IgM) concentration, and frequent concomitance with other autoimmune diseases such as Hashimoto’s thyroiditis and autoimmune hepatitis (AIH) [1,2,3]. However, AMA is highly specific with a pattern of being positive in approximately 90–95% of patients with PBC [4]. The etiology of PBC includes genetic susceptibility and environmental factors, specific geography, and family history, but is to date not fully understood [4,5,6,7]. Its incidence ranges between 0.9 and 5.8 per 100,000 population per year, while its prevalence is 1.9–40.2 per 100,000 [1,2,8]. Similarly to other autoimmune diseases, PBC has witnessed a constant increase in the last two decades in Western countries [1,9].

With regard to physiology, bile is synthesized in the hepatocytes, then secreted and altered when passing through the biliary tree by the epithelial cells lining the bile duct (cholangiocytes) [10]. The bile has the following main functions: digestion and absorption of fat and fat-soluble vitamins, excretion of bilirubin and cholesterol, neutralization of the acidic pH from the stomach, and provision of bacterial activity against microorganisms [10]. From a pathogenetic point of view, the impaired bile flow in PBC leads to the accumulation of bile acids, thereby causing inflammation to biliary epithelial cells (“cholestatic hepatocyte injury”), increased periportal inflammation, and progressive fibrosis of the liver parenchyma [11,12,13]. Liver cirrhosis is the clinical endpoint of this (often silent) process lasting for years and increasing morbidity and mortality dramatically, which is mostly caused by portal hypertension-induced complications and hepatocellular carcinoma. Liver fibrosis (and even cirrhosis) seem to be reversible as long as a certain “point of no return” is not exceeded. Therefore, it is of fundamental importance to assess the degree of actual liver damage, to determine its etiology, and to initiate adequate treatment to prevent ongoing liver damage [14].

PBC and primary sclerosing cholangitis (PSC) are currently the main types of intrahepatic cholestatic liver disease [15]. While elevated blood levels of bilirubin, alkaline phosphatase (AP), and gamma-glutamyl transferase (GGT) are the laboratory hallmarks of cholestasis, hepatic inflammation usually comes along with increased levels of transaminases, e.g., aspartate aminotransferase (AST) and alanine aminotransferase (ALT). The elevation of AP and GGT, after ruling out other causes of liver injury such as alcoholism or drug-induced liver injury, strongly suggests damage to cholangiocytes, indicating the presence of cholestatic liver disease [15,16].

Besides biopsy, liver fibrosis can be assessed by ultrasound-based methods such as transient elastography (TE; Fibroscan). By the use of TE, the stiffness of the liver tissue can be measured by low-frequency vibrations, which is supposed to be proportional to the extent of fibrosis [16,17]. More recently, the measurement of the enzymatic liver function by the liver maximum capacity (LiMAx) breath test has been introduced as a robust technique to determine liver function based on the specific hepatic cytochrome p 450 1A 2 metabolism of an intravenously injected substrate [16,18]. In a prospective study by our group, we could demonstrate that enzymatic liver function measured by LiMAx was closely associated with histologically proven parenchymal changes (fibrosis) in 102 patients with chronic liver disease (CLD) [14]. In addition, LiMAx was successfully evaluated in various clinical situations [16,19,20,21,22].

Another promising alternative for the early detection of hepatic diseases is liquid biopsy. A liver liquid biopsy is a minimally invasive test that measures liver-originated by-products, such as proteins, circulating tumor cells, and (micro-)RNA in plasma [23]. Liquid biopsies are supposed to precede morphological liver changes during the progression/regression of a benign or malignant liver disease process and were evaluated in, for example, metabolic dysfunction-associated steatohepatitis (MASH), hepatocellular carcinoma, and pancreaticobiliary cancer [24,25,26].

The treatment paradigm for PBC is mainly based on bile acids and peroxisome proliferator-activated receptor (PPAR) ligands [1,27]. The introduction of ursodeoxycholic acid (UDCA), a physiological component of human bile acids at low concentrations (<3% of total bile acids), in the treatment of patients with PBC has changed the disease course and is the recommended first-line therapy [13]. In this regard, multiple randomized controlled trials and meta-analyses could demonstrate that UDCA significantly improves the biochemical response, delays histological progression, and prolongs the transplant-free survival of patients suffering from PBC [15,28,29,30]. Currently, the life expectancy in patients with PBC with a response to UDCA therapy is supposed to be similar to patients without PBC [1]. However, up to 40% of patients with PBC have a suboptimal response (failure to achieve effective biochemical improvement) or intolerance to UDCA treatment, necessitating second-line therapies [31]. Fortunately, therapeutic options evolved during the last decades and a bouquet of drugs have become available for UDCA non-responders focusing on individual decision making and personalized therapy. For second-line treatment of patients with PBC, the current guidelines recommend obeticholic acid (Ocaliva; OCA), budesonide, and fibrates. However, budesonide and fibrates are currently used as off-label therapy [12,13,15,32]. OCA is a semi-synthetic bile acid analog and a Farnesoid X receptor (FXR) agonist which inhibits the synthesis of bile acids. Several studies have shown that for patients with poor response to UDCA, adding or switching to OCA (5–10 mg/kg/d) significantly improves biochemical response and delays the histological progression of patients with PBC [12,15,33].

In this prospective pilot study, between 09/2022 and 09/2023, we included 16 non-cirrhotic patients with a confirmed diagnosis of PBC and insufficient response to UDCA therapy who were treated with add-on OCA (5 mg/d). We therefore focused on the six-month short-term biochemical, structural, and functional response to OCA treatment.

## 2. Patients and Methods

### 2.1. Patient Information, Data Collection, and Ethical Considerations

A total of 16 adult patients treated at the University Hospital of Essen were included in this prospective pilot study between September 2022 and September 2023. None of the involved patients suffered from concomitant AIH. The study was conducted according to the principles expressed in the Declaration of Helsinki and was approved by the local ethic committee (16-7228-BO). Informed consent was available for all patients included in the study.

### 2.2. Drug Treatment, Definition of UDCA Non-Response, and Laboratory Parameters

Patients included in this prospective pilot study had a confirmed diagnosis of PBC. Diagnosis was based on persistent cholestatic abnormalities in serum liver tests (abnormal serum level of AP, GGT, serum bilirubin), increased IgM concentrations, AMA, and specific antinuclear antibody (ANA) reactivity according to actual EASL recommendations [13]. Liver biopsy was only performed in uncertain cases (e.g., AMA negativity) or in cases with a suspected overlap to AIH or MASH. None of the patients had an overlap to AIH. At the timepoint of study inclusion, all patients were on drug therapy with UDCA (13–15 mg/kg/d) for at least six months without adequate biochemical response (normalization of AP) according to the actual guidelines [13,34]. All patients were treated with a dosage of 5 mg OCA per day. The determination of laboratory parameters (e.g., AST, ALT, AP, and GGT) and the performance of Fibroscan and LiMAx measurements were carried out at the timepoint of study inclusion and at the 6-month follow-up examination. Patients with (decompensated) liver cirrhosis were not included in this study.

### 2.3. Liver Maximum Capacity (LiMAx) Test

The LiMAx test (Humedics, Berlin, Germany) was performed after a minimum of 3 h fasting. The measurement is based on the hepatocellular-specific metabolism of intravenously administered ^13^C-labeled methacetin—an exclusive substrate for the hepatic cytochrome P450 1A2 enzyme. ^13^C-methacetin in hepatocytes is immediately demethylated into acetaminophen and ^13^CO_2_; the latter is subsequently exhaled, leading to an increase in ^13^CO_2_ concentration in breath. Prior to substrate injection, the individual baseline ratio ^13^CO_2_/^12^CO_2_ concentration of a patient is measured, and thus, the liver function capacity can be calculated from the analysis of the ^13^CO_2_/^12^CO_2_ ratio within 60 min after injection. Results are given in μg/kg/h [20].

### 2.4. Liver Stiffness Measurement (LSM) by Transient Elastography (TE)

LSM (Fibroscan, Echosens, Paris, France) was performed after fasting for at least 8 h pre-exam and with the patient in a supine position with the right arm in maximal abduction. The standard TE probe (type M) was used via intercostal spaces on the right lobe of the liver. In patients with obesity, the XL-probe was applied. Each examination was assisted by B-mode ultrasound (APLIO 500, Toshiba, Japan) for the identification of a feasible probe position and exclusion of perihepatic ascites. The median of at least 10 LSM values expressed in kilopascal (kPa) was used as the representative measurement. The success rate was calculated as the number of valid measurements divided by the number of total measurements. According to the manufacturer’s recommendation and published evidence, only patients with an interquartile range (IQR) < 30% of the median value and a success rate > 60% were included in the analysis [20,35]. LSM was performed by an experienced observer who had performed at least 500 examinations each.

### 2.5. Statistical Analysis

Statistical analyses were performed to assess the significance of changes in paired values. Initially, the normality of the data distribution was evaluated using the Shapiro–Wilk test. If the data followed a normal distribution (*p* > 0.05), the paired *t*-test was applied to compare differences between groups. However, when the assumption of normality was violated (*p* ≤ 0.05), the non-parametric Wilcoxon signed-rank test was used. Statistical significance was set at *p* < 0.05. The values are demonstrated in median and interquartile range (25% percentile; 75% percentile).

## 3. Results

### 3.1. Demographic Data

A total of 16 adult patients with PBC with insufficient response to UDCA were included in this pilot study, with 14 of Caucasian and 2 of Asian origin. Most of the study population was female (15/16, 93.75%), and the mean age was 57.63 ± 9.59 (43–77) years.

### 3.2. Course of Laboratory Parameters During OCA Treatment

**Figure 1:**
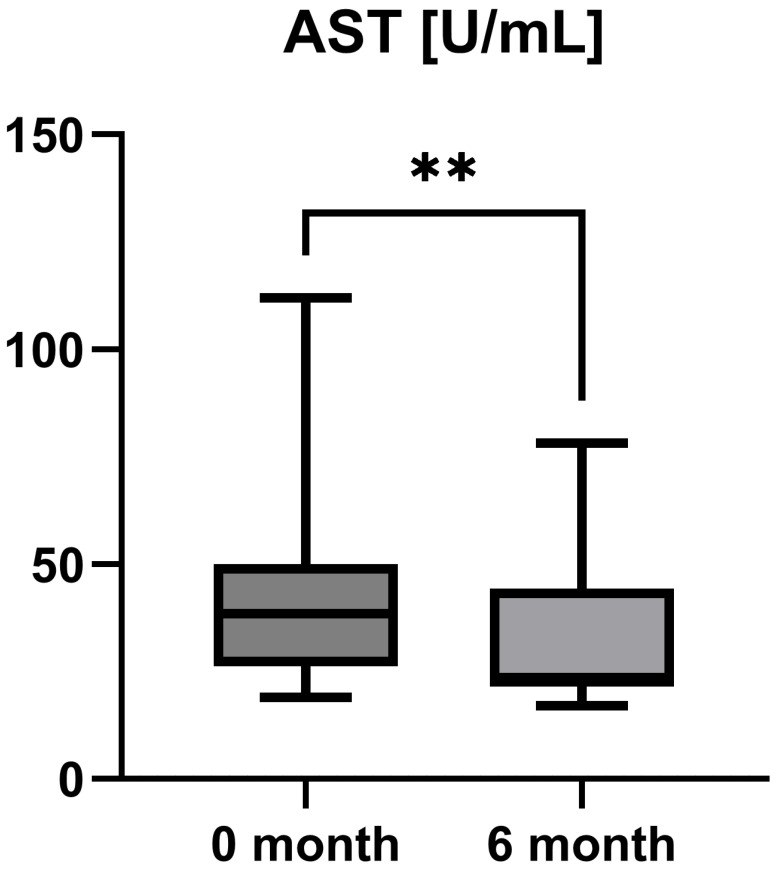
Course of individual aspartate aminotransferase (AST) values during OCA treatment (*p* = 0.0012). ** *p* < 0.01.

**Figure 2:**
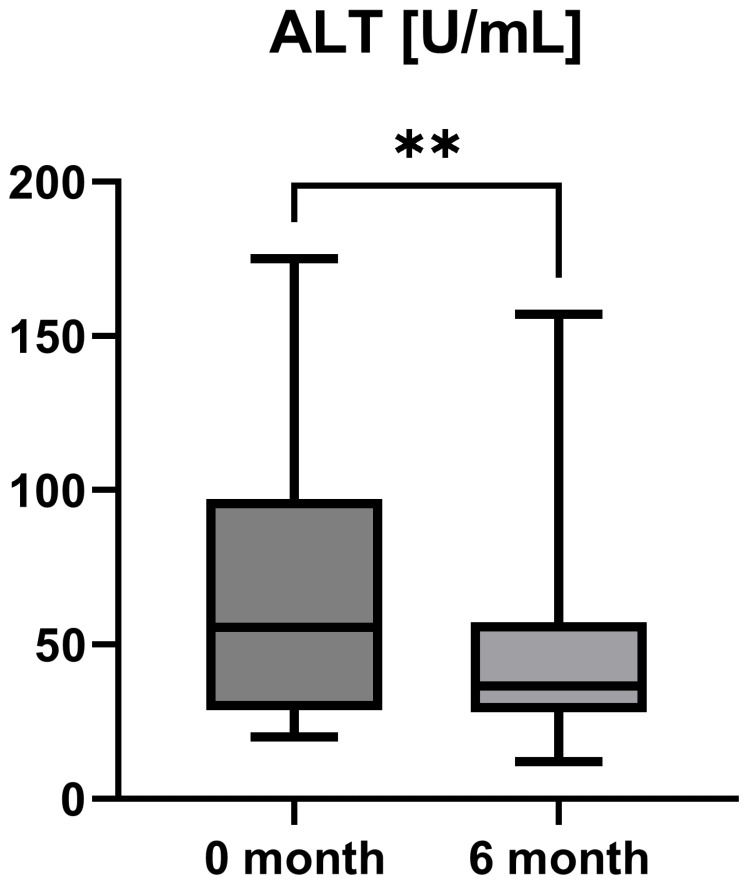
Course of individual alanine aminotransferase (ALT) values during OCA treatment (*p* = 0.0035). ** *p* < 0.01.

**Figure 3:**
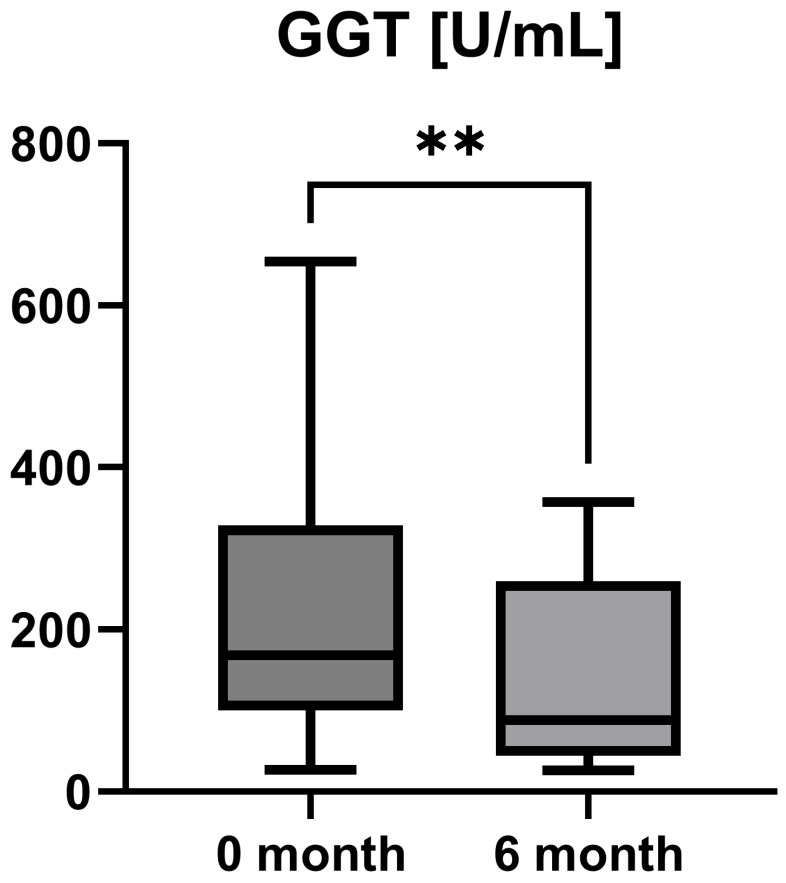
Course of individual gamma-glutamyl transferase (GGT) values during OCA treatment (*p* = 0.0063). ** *p* < 0.01.

**Figure 4:**
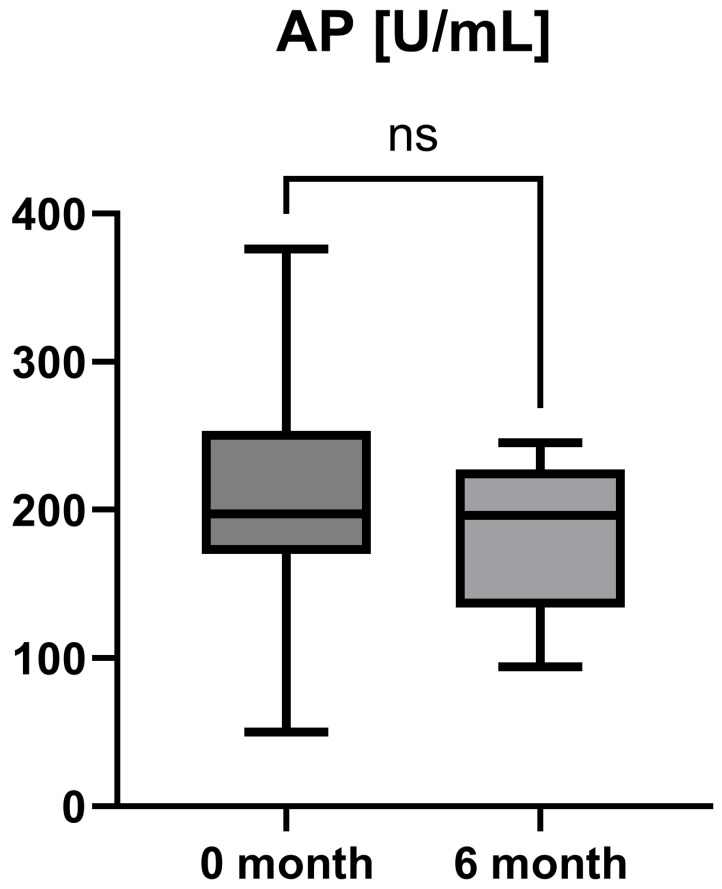
Course of individual alkaline phosphatase (AP) values during OCA treatment (*p* = 0.0915). ns = not significant.

Laboratory parameters such as transaminases and parameters of cholestasis (e.g., AST, ALT, AP, and GGT) were determined at the timepoint of study inclusion (before the start of add-on therapy with OCA) and at the 6-month follow-up examination. However, OCA treatment led to a statistically significant decrease in AST (AST baseline: 38.50 [26.25; 50.00] IU/L vs. AST 6-month follow-up: 23.50 [21.50; 44.25] IU/L, *p* = 0.0012), ALT (ALT baseline: 55.50 [28.75; 97.00] IU/L vs. ALT 6-month follow-up: 36.50 [28.00; 57.25] IU/L, *p* = 0.0035), and GGT (GGT baseline: 168.00 [100.30; 328.50] IU/L vs. GGT 6-month follow-up: 88.00 [44.50; 259.80] IU/L, *p* = 0.0063), while the decrease in AP was not statistically significant (AP baseline: 197.00 [170.00; 253.30] IU/L vs. AP 6-month follow-up: 196.00 [134.00; 227.00] IU/L, *p* = 0.0915). The course of liver enzymes during OCA treatment is demonstrated in Figure 1, Figure 2, Figure 3 and Figure 4.

### 3.3. Course of Liver Stiffness Measured by Fibroscan and Liver Function Measured by LiMAx During OCA Treatment

**Figure 5:**
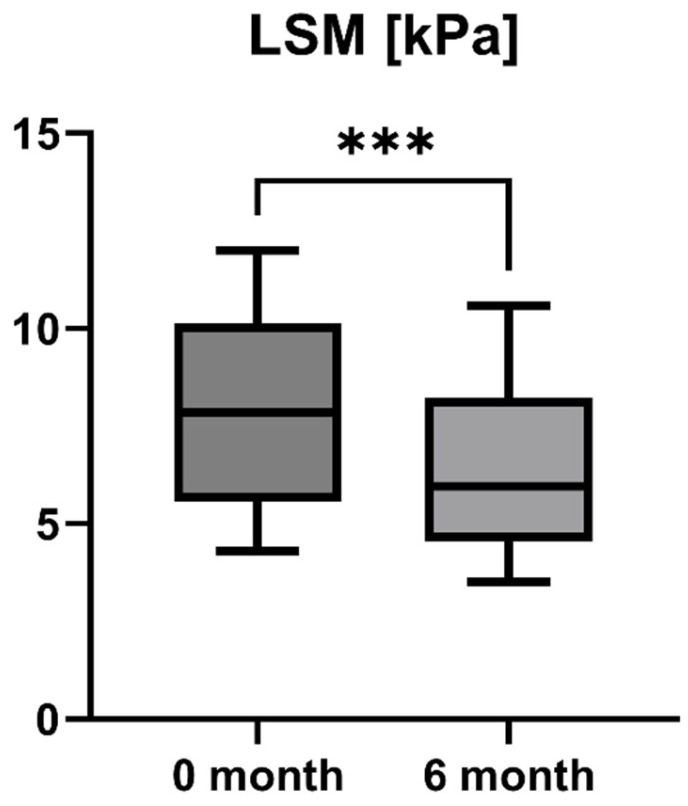
Course of individual liver stiffness measurement (LSM) values during OCA treatment (*p* = 0.0001). *** *p* < 0.001.

**Figure 6:**
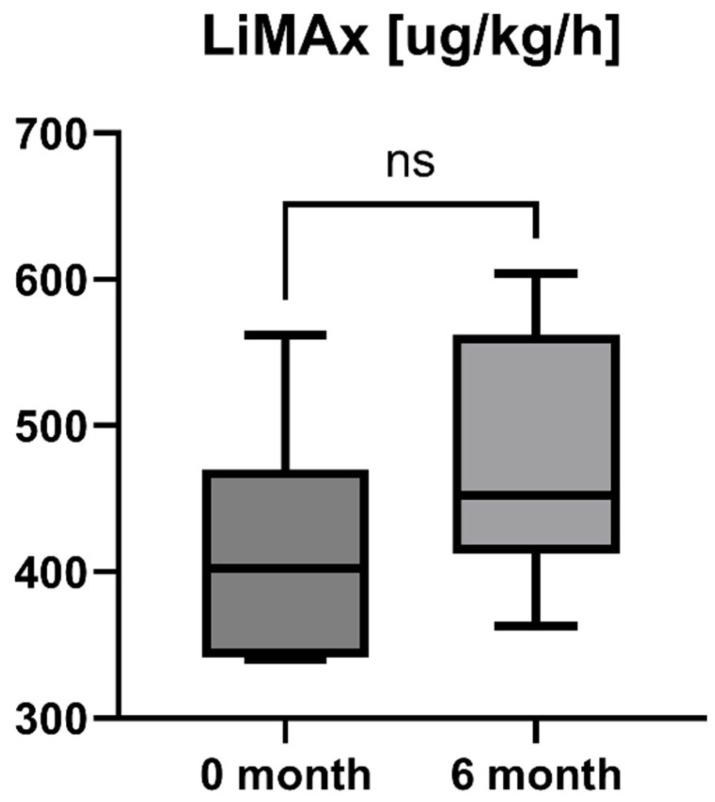
Course of individual LiMAx values during OCA treatment (*p* = 0.0625). ns = not significant.

Accordingly, the liver stiffness measured by Fibroscan was performed at study inclusion and 6-month follow-up. LSM was available for all patients (*n* = 16) and showed a statistically significant decrease after six months of treatment with OCA (LSM baseline: 7.85 [5.55; 10.13] kPa vs. LSM 6-month follow-up: 5.95 [4.55; 8.225] kPa, *p* = 0.0001). However, the increase in enzymatic liver function, as measured by LiMAx, failed to reach statistical significance, but showed a positive trend (LiMAx baseline: 402.50 [341.50; 469.80] μg/kg/h vs. LiMAx 6-month follow-up: 452.50 [412.50; 562.00] μg/kg/h, *p* = 0.0625). Unfortunately, valuable LiMAx measurements were only available for 6/16 patients (37.5%) at both timepoints. The course of liver stiffness measured by Fibroscan and the liver function measured by LiMAx are shown in Figure 5 and Figure 6.

### 3.4. Side Effects of OCA Treatment and Discontinuation of Treatment

Overall, OCA treatment was well tolerated by the study population. Three patients reported a new onset or worsening of pre-existing pruritus after the initiation of therapy. If necessary, pruritus was treated with second-generation antihistamine cetirizine 10 mg per day (administration preferably in the evening). However, in none of the patients did OCA therapy have to be discontinued due to clinically relevant side effects.

## 4. Discussion

PBC is a chronic autoimmune, cholestatic, and slowly progressive disease that can lead to cirrhosis and liver failure in the case of delayed diagnosis and inadequate treatment [1,2,11,12,13]. According to our cohort (93.75% females, mean age 58 years), PBC mainly affects adult women aged 40–70 years [1,2,3,36].

Besides female gender, genetic risk factors likely play an important role in conferring susceptibility to PBC, as indicated by the high concordance rates among monozygotic twins and first-degree relatives of affected individuals [37,38]. However, environmental factors (for example, infections) are supposed to break immunological tolerance and lead to the onset of PBC in genetically susceptible individuals [39,40]. Although there exists no causal therapeutic approach, pharmacological therapies and lifestyle changes such as following the Mediterranean diet and avoiding alcohol and tobacco consumption, can halt the progression to cirrhosis and/or liver failure, and alleviate symptoms associated with the disease [41]. The clinical course of patients with PBC might be variable ranging from mild forms without complications to severe cholestatic disease with jaundice, portal hypertension and decompensation. The mainstay of PBC treatment focuses on therapeutic approaches which on the one hand positively influence the course of the disease (e.g., UDCA, OCA, fibrates) and on the other hand improve associated symptoms, for example, pruritus (e.g., colestyramine, rifampicin, sertraline). We are therefore convinced of individualized clinical pathways for patients with PBC.

Currently, UDCA, at a target dosage of 13–15 mg/kg per day, represents the standard-of-care therapy for patients suffering from PBC improving inflammatory activity, cholestasis, disease progression, and survival [15,28,29,30]. However, larger doses of UDCA (28–32 mg/kg/d) do not increase the clinical benefits [13,15,42]. Unfortunately, not all patients respond to UDCA since cholestasis and hepatic inflammation persists despite therapy [43]. Possible mechanisms for UDCA non-response include cellular senescence, immune-mediated damage, and vitamin D deficiency [44]. Several criteria for UDCA treatment have been developed to evaluate patient risk stratification, such as Rotterdam, Barcelona, Paris I/II, Toronto, GLOBE, and UK-PBC [45,46,47,48,49,50]. These prognostic models have in common the assessment of the therapeutic effects using biochemical parameters after UDCA treatment initiation for 6, 12, or 24 months, while the 12-month period is conventionally used to identify patients in needs for second-line therapies [13]. Compared to responders to UDCA, these patients are at greater risk of hepatic complications such as ascites, variceal bleeding, and hepatic encephalopathy [51,52]. However, the above mentioned response criteria pose potential limitations for patients with inadequate response who are at higher risk for disease progression to receive non-effective treatment for a long period [53]. Furthermore, although the safety profile of UDCA is generally satisfactory, clinically relevant side effects such as pruritus, diarrhea, abdominal pain, and urticaria sometimes restrict its use [15,54,55].

More recently, the approval of the FXR agonist OCA in early 2017 expanded the therapeutic options of PBC. In contrast to other drugs applied in patients with PBC, OCA is the only substance which has officially been approved by EASL and AASLD for the second-line treatment of patients with PBC [12,13,15,32]. The phase III study of OCA in patients with PBC (POISE) registration study involved 216 patients who did not respond adequately to UDCA treatment or could not tolerate UDCA treatment. Patients were treated with OCA 5–10 mg/d or placebo. Therapeutic response, defined as reduction in alkaline phosphatase (AP) < 1.67 × ULN and concomitant reduction in AP of >15% from pre-treatment levels and normalization of serum bilirubin, was achieved by 47% in the OCA group and 10% in the placebo group (*p* < 0.001) after 12 months of treatment and remained in both groups after 24 months of treatment [56]. In the meantime, other clinical trials and meta-analyses could validate the effectiveness of OCA through the adjustment of biochemical and histological findings as well as the improvement of survival of patients with PBC [57,58,59,60]. More recently, Brookhart et al. identified a 63% reduced risk of hospitalization for hepatic decompensation, liver transplant, and death in OCA-treated versus non-OCA-treated individuals in the HEROES trial [61]. In 2021, the U.S. Food and Drug Administration (FDA) issued a new warning restricting the use of OCA in patients with decompensated cirrhosis (Child–Pugh class B or C) or a previous event of decompensation, and in patients with compensated cirrhosis who have evidence of portal hypertension [36,62]. This recommendation is, among others, based on the results of a study of Londono et al. in which 10 from 25 cirrhotic patients with portal hypertension decompensated during treatment with OCA (four had to undergo liver transplantation, two died) [63]. However, none of our patients had clinical, laboratory, or imaging signs of liver cirrhosis or portal hypertension, and baseline TE measurements were below the cut-off for advanced fibrosis/cirrhosis (12.5 kPa) in all patients. In 2024, the European Medicines Agency (EMA) recommended revoking conditional approval for OCA based on the partially enrolled results of the COBALT trial in which OCA failed to show superior outcomes compared to placebo [64]. However, critics have cited difficulties in the study design and execution of the COBALT trial, which actually led to the withdrawal of the EMA’s recommendation. In this regard, the last word does not seem to have been spoken yet.

PPAR agonists (fibrates) signal via specific intranuclear receptors to control diverse metabolic processes, including lipid and bile acid metabolism [15,27]. Fibrates are widely licensed for the treatment of dyslipidemia [65]. In addition, PPAR agonists, such as bezafibrate and elafibranor, downregulate the expression of bile acid synthase CYP7A1, which is the key regulator of bile acid synthesis, thereby having beneficial effects on PBC in (pre)clinical trials [36,56,66]. With regard to the autoimmune nature of PBC, the synthetic corticosteroid budesonide, which is metabolized during the first hepatic pass, showed biochemical improvement, but failed to improve histological findings in patients with PBC [36,42]. Due to insufficient evidence, fibrates and corticosteroids are currently used as off-label therapy options in patients with PBC.

In the present pilot study, we included 16 patients with a confirmed diagnosis of PBC and inadequate response to UDCA who were treated with an add-on therapy with OCA 5 mg/d. Besides biochemical routine parameters (e.g., AST, ALT, AP, and GGT) and liver stiffness (determined by TE), enzymatic liver function (measured by the novel LiMAx test) was determined to enable an additional individual differentiation at the timepoint of study inclusion (before start of add-on therapy with OCA) and at 6-month follow-up examination. Since these diagnostic parameters and techniques are dynamic, they do not only reflect the actual status but can also serve for monitoring patients with CLD, especially with regard to success or failure of pharmacological therapies.

OCA as an FXR agonist has a pluripotent mode of action in PBC, regulating bile-acid conjugation and its transport, reducing liver inflammation (as shown by liver enzymes), and, finally, diminishing fibrosis (as demonstrated by TE and LiMAx analyses in a short-term clinical course). These improvements showed statistical significance (*p* < 0.05) for all examined parameters except AP (*p* = 0.09) and LiMAx (*p* = 0.06), in which a positive trend could be documented (Figure 1, Figure 2, Figure 3, Figure 4, Figure 5 and Figure 6). GGT and AP represent cholestatic hepatic injury in blood. However, AP failed to reach statistical significance, which might be explained by (i) the higher sensitivity of GGT for diagnosing cholestasis, (ii) the fact that AP is not exclusively expressed in hepatocytes but is also expressed in the bones (lower specificity), and (iii) the small sample size of the study cohort.

Possible side effects may also hamper the ability of OCA treatment. A meta-analysis including patients suffering from PBC and metabolic-associated steatohepatitis treated with OCA showed that OCA increased the risk of pruritus by about 75% and the risk of constipation by about 88% [11,67]. In addition, pruritus was reported as an adverse effect in 56–58% of patients receiving OCA in the POISE [56]. Our study shows that pruritus can currently be managed effectively and does not lead to treatment discontinuation. Overall, in none of our study patients did OCA have to be withdrawn due to side-effects during the observation period.

As discussed above, the landscape of therapeutic options in patients with PBC have expanded over last decades. This is important since a significant proportion of patients with PBC do not respond adequately to UDCA therapy. However, an adequate biochemical response to pharmacological treatment is the key factor in the clinical course for patients with PBC to prevent disease progression to end-stage liver disease and liver failure, thereby reducing the need for liver transplantation in times of organ shortage [68].

Fortunately, different treatment modalities have become available, and the management of patients with PBC is becoming more and more individualized. Besides OCA, PPAR agonists will expand the therapeutic options for patients with PBC as promising alternatives “ante portas”. The awareness of the disease and adequate therapy should be raised since the recently published results of the German PBC registry indicate suboptimal therapy in a significant proportion of patients [69].

We are aware of the limitations of our study, the most important of them being the small cohort size, the short observation period, and the fact it is a single-center study. We still believe that these prospective data are of high clinical importance, since they emphasize the effectiveness of OCA for patients who have not responded to UDCA therapy. However, larger, multi-center studies with a longer observation period are warranted to confirm these results.

## 5. Conclusions

In conclusion, we demonstrated that OCA is a reasonable adjuvant therapy in non-cirrhotic patients with PBC with inadequate response to UDCA. To the best of our knowledge, this is the first study showing biochemical, structural, and functional improvement of PBC after switching to OCA treatment in a short-term clinical course.

## Data Availability

The raw data supporting the conclusions of this article will be made available by the corresponding author on request.

## Abbreviations

AIH: autoimmune hepatitis; ALT, alanine aminotransferase; AMA, antimitochondrial antibody; ANA, antinuclear antibody; AP, alkaline phosphatase; AST, aspartate aminotransferase; CLD, chronic liver disease; FXR, Farnesoid X receptor; GGT, gamma-glutamyl transferase; IgM, immunoglobulin M; LiMAx, liver maximum capacity; LSM, liver stiffness measurement; MASH, metabolic dysfunction-associated steatohepatitis; OCA, obeticholic acid; PBC, primary biliary cholangitis; PPAR, peroxisome proliferator-activated receptor; TE, transient elastography; UDCA, ursodeoxycholic acid.
